# Case of a Large Dose of Laudanum Proving Fatal, Although Full Vomiting Was Speedily Effected

**Published:** 1814-03

**Authors:** James Cornish

**Affiliations:** Member of the College of Surgeons


*For the Medical and Physical Journal.*

### *Case of a large Dose of Laudanum proving fatal, although full Vomiting was speedily effected.* By Mr. JAMES CORNISH, Member of the College of Surgeons.

WHEN opium is taken in large quantities, either by mistake, or with an intention of destroying life, we are directed to attempt a speedy evacuation of the poison, and to endeavor to counteract its effects.

In cases where mineral poisons have been swallowed, there seems good ground to hope, that by the judicious management of well-chosen remedies, this latter indication may be fulfilled, and the danger arising from their exhibition guarded against and prevented. As the vegetable poisons, however, do not admit of such remedies, from our not possessing the means to render their deleterious qualities inert by decomposition, and as their narcotic property will in a short time so far destroy the sensibility of the stomach, and induce such a degree of spasm as to render vomiting impracticable; their speedy evacuation is the indication on which we have principally to rely.

It would appear to have very generally obtained, from some successful cases which have been published, that where free vomiting can be procured, but little danger is to be apprehended. Such, I frankly confess, was my opinion as to the result of the following case; and my motive in requesting its insertion in the Medical Journal is, that in similar cases a like disappointment may not be experienced.

John Bunny, ætat. 75, Saturday, Jan. 8, at eight, a. m. swallowed, according to his own confession, an ounce and a half half of tincture of opium. From the account his wife gave me, I supposed he must have taken it half an hour, rather less than more, when I first saw him. He was sitting upright in a chair, apparently in the perfect possession of his senses as he had been for some days previously. His mind, however, had been irritated by some circumstances that had occurred, and his friends had observed some peculiarities in his conduct which had led them to consider him as imperfectly master of his reason. An emetic* of ten grains of tartrate of antimony and half a dram of ipecacuanha was given him without delay, which in the space of fifteen minutes brought from his stomach nearly a wine glass full of fluid, in which opium was strongly perceptible. The emetic operated three or four times in the course of the morning, and as so short a period had elapsed between its exhibition and the taking the poison, together with the consideration that the greatest part, if not *all*, of the offending matter must have been rejected, very little apprehension was entertained respecting the result of the case. The narcotic effect of the opium, however, soon became apparent, his pulse became slow, his breathing deep and sonorous, and his inclination to sleep very considerable. When roused he answered rationally. His drowsy disposition was prevented by continually shaking him, and by obliging him to walk the room, which he did for some time with very little assistance; but as he gradually lost the use of his legs, and as his insensibility increased, it was necessary to again seat him in a chair. Strong coffee, and diluted acetic acid, were plentifully given him, and by now and then irritating the throat with a feather, he vomited freely. In what he rejected after the first four or five times, the smell of the laudanum was not to be perceived. Notwithstanding these free evacuations, his breathing continued stertorous, his powers of speech and voluntary motion failed him, and his inclination to sleep was almost invincible. Violently shaking him, volatile alkalies to the nostrils, blisters to the back, frictions with salt, and irritating the glottis, with difficulty roused him. In this state he continued till the evening, when he regained the faculty of speech, but soon after relapsed into a comatose state. Clammy sweats broke out, his pulse became indistinct and intermittent, and his nervous energy almost exhausted, when a full dose of carbonate of ammonia was given him; he was also directed to take a small quantity of diluted spirit occasionally. These

* The tartrate of antimony was given, as it was on the spot, and that no time might be lost. The sulphate of zinc would have been preferred could it have been instantly procured.

stimuli proved of temporary good effect. The patient was kept in a state of forced activity till towards the following morning, when he was suffered to remain three or four hours undisturbed; after this he was awoke and seated in a chair, and a purgative draught was administered. The stupor and insensibility continued throughout the day; his hands were cold, and his pulse feeble, irregular, and intermitted at every fifth beat. In the evening he regained his senses so far as to be able to speak, and he walked his chamber with little difficulty. He also made several attempts to empty his bladder, but was unable to excite it to contract. The draught not having produced the desired effect, a stimulating clyster was advised, but from his being restless it could not be administered; the draught was therefore repeated. A short time after, as on the preceding evening, he relapsed into a state of insensibility, and continued in this state throughout the night. By the morning his pulse was scarcely to be felt, his eyes appeared sunk, and his countenance ghastly. Thus he remained till three, p. m. when his vital powers ceased to perform their functions.

The following day, with the assistance of my friend Mr. Doherty, whose anatomical knowledge I feel pleasure in noticing, an examination was made of the contents of the cranium, thorax, and abdomen, more from motives of anatomical curiosity than from an expectation of discovering novel appearances from the effect of a narcotic poison.

On removing the skull-cap, the vessels of the dura mater were observed much loaded, and coagulable lymph in considerable quantity was effused between the dura mater and tunica arachnoides. On attempting to raise the dura mater alone, it was found impracticable; the connection with the tunica arachnoides by means of the lymph being so strong; and the effusion also existing between the arachnoid coat, and pia mater, the three membranes were raised together as if they had been naturally united. With the exception of the lateral ventricles containing more fluid than is commonly the case, there was nothing further remarkable in the cavity of the cranium.

The semilunar valves of the heart were ossified, as was also the origin of the coronary artery. The mitral valves were in a natural condition. There was nothing preternatural in the appearance of the stomach. The corrugation, and slight inflammation which in similar cases have been observed, were not to be seen in this instance. It contained panada, &c. (what he had last taken) in an unaltered state. The presence of opium could not be detected. The spleen was unusually small, corrugated, and gritty on its surface.

 From

From the intemperate life this man had led, a healthy condition of the liver was not expected; it was however of its natural size, color, and consistence. The bladder contained about a pint of urine. The opium appeared to have deprived this viscus of the power of contraction.

*Falmouth, Feb.* 4, 1804. JAMES CORNISH.

